# Hippocampal Atrophy Following Therapeutic Irradiation of Intracranial and Nasopharyngeal Tumors

**DOI:** 10.7759/cureus.98783

**Published:** 2025-12-09

**Authors:** Mikhail V Galkin, Andrey V. Golanov, Natalia Antipina

**Affiliations:** 1 Department of Radiation Oncology, N.N. Burdenko National Medical Research Center of Neurosurgery, Moscow, RUS

**Keywords:** brain neoplasms, dose-response relationship, hippocampal atrophy, hippocampus volume, nasopharyngeal cancer (npc), neurotoxicity, radiation-induced cognitive impairment, radiotherapy, volumetry, whole-brain radiotherapy

## Abstract

The review aims to summarize and evaluate existing data on macroscopic changes in hippocampal volume following therapeutic brain irradiation, with a focus on the patterns, dynamics, and modulating factors of this structural damage. This narrative review is based on a structured search of the PubMed/Medical Literature Analysis and Retrieval System Online (MEDLINE) database and relevant bibliographies (2000-2025), selecting studies that applied MRI volumetry to assess hippocampal changes after radiotherapy. We summarized the results of studies using MRI volumetry in patients undergoing radiation therapy for central nervous system tumors and nasopharyngeal cancer. The analysis covered various irradiation methods, including local and whole-brain techniques, with a focus on dose-volume relationships, temporal dynamics, and differential vulnerability of subfields. The data indicate a marked, dose-dependent reduction in hippocampal volume following irradiation. The degree of atrophy, often reaching 5-9% in the early years, significantly exceeds normal age-related decline and may rival that seen in neurodegenerative pathologies. Certain subregions, particularly the dentate gyrus and CA1, show particular sensitivity. The temporal development of atrophy varies, showing both rapid initial loss followed by stabilization and progressive patterns. The pediatric population may show partial recovery of the growth trajectory, albeit with a reduced baseline. Critical factors influencing the degree of atrophy include patient age and, potentially, neoadjuvant chemotherapy, while concurrent chemotherapy is often not a determining factor. Quantitative assessment of hippocampal volume change is a reliable and objective biomarker of radiation-induced neurotoxicity. This parameter is an important tool for refining hippocampal radiation dose constraints in radiation therapy planning and for functional assessment after treatment, highlighting the need to incorporate hippocampal protection strategies into modern neuro-oncology practice.

## Introduction and background

Radiation therapy (RT) is a cornerstone treatment for tumors [1,2]. It is widely used in oncology, including neuro-oncology, for managing various benign and malignant tumors, as well as certain non-neoplastic conditions. Given the complex anatomy and functional significance of neural structures, RT is particularly important in neuro-oncology. It is frequently employed not only as an adjuvant treatment but also as a primary therapeutic modality.

The physical properties of ionizing radiation, along with the radiobiological characteristics of different tissues, influence the risk of damage to structures located within or near the irradiation target [3,4]. This risk determines the probability of complications arising from therapeutic radiation.

Recently, significant interest has emerged in studying the effects of radiation on the hippocampus, particularly potential morphological and neuropsychological changes [3-6]. Hippocampal damage is considered a key factor in the development of cognitive impairment in patients receiving RT for primary and secondary CNS tumors, as well as head and neck tumors. This is supported by extensive direct and indirect laboratory and clinical evidence.

A comprehensive review by Gondi and colleagues details the cognitive consequences of radiation exposure and the role of the hippocampus in this process [5]. The review by Son et al. presents data on the multifaceted negative effects of RT on the hippocampus, including suppressed neurogenesis, trophic disturbances, ischemic changes, and chronic inflammation [6].

Several lines of evidence confirm the key role of radiation-induced hippocampal damage. Animal studies demonstrate morphological changes at the micro-level, impaired neurogenesis in the hippocampus, and volume reduction following irradiation [7,8]. Animal research has also shown that localized hippocampal irradiation impairs cognitive function [9]. This is accompanied by a decrease in the proliferative activity of immature neurons in the subgranular zone of the dentate gyrus. Since memory formation is associated with stem cell proliferation in the hippocampal subgranular zone, this impairment is particularly significant. After whole-brain radiotherapy (WBRT), the hippocampus volume decreases significantly more than that of white matter or the cortex [10]. Autopsies of patients who previously received craniospinal irradiation (CSI) revealed a substantial (10-fold) decrease in neurogenesis, even years after exposure [11]. Functional magnetic resonance imaging (fMRI) data indicate altered functional connectivity of the hippocampus in patients after RT [12]. Numerous studies have demonstrated a dose-dependent relationship between hippocampal radiation exposure and the development of cognitive impairment [13-16].

These cognitive sequelae have prompted efforts to limit the radiation dose to the hippocampus, leading to the development of WBRT with hippocampal avoidance (HA-WBRT), which significantly reduces the risk of cognitive decline [17]. Several dose constraints have been proposed. One constraint specifies that the dose to 40% of the volume of both hippocampi (D40%) should not exceed 7.3 Gy (equivalent dose in 2 Gy fractions (EQD2), α/β=2) [13]. Other protocols, for a regimen of 10 fractions of 3 Gy, recommend that the dose to 100% of the hippocampus (D100%) should not exceed 9 Gy (EQD2 6.5 Gy, α/β=2), and the maximum dose (Dmax) should not exceed 16 Gy (EQD2 14.4 Gy, α/β=2) [15,17]. Another set of constraints proposes D98% ≤ 9 Gy (EQD2 6.2 Gy, α/β=2), D2% ≤ 17 Gy (EQD2 14.5 Gy, α/β=2), and a goal for the mean dose (Dmean) ≤ 10 Gy (EQD2 7.1 Gy, α/β=2) [18].

This narrative review focuses on the macroscopic, MRI-detectable hippocampal volume loss (atrophy) following therapeutic radiation exposure. We aim to synthesize the existing literature on volumetric changes, providing a resource for clinicians and researchers. The scope is specifically limited to gross volumetric changes; microstructural alterations assessed by techniques such as diffusion MRI, as well as functional or metabolic changes, are beyond the scope of this synthesis. Specifically, it examines how hippocampal volume changes after RT and how this varies depending on radiation type, dose, patient age, timing, and other conditions. The reviewed studies typically include the following patient groups: adults after local RT for CNS tumors, local RT for head and neck tumors, or WBRT, and children after WBRT, sometimes with an additional local "boost". The treatment regimens for these conditions differ substantially in terms of total dose, fractionation, and irradiated volume. Consequently, the hippocampi may be exposed to a wide spectrum of radiation doses, from high-dose focal RT to moderate-dose exposure during whole-brain treatment. 

## Review

Materials and methods

Search Strategy and Selection Criteria

This narrative review was conducted in accordance with Preferred Reporting Items for Systematic Reviews and Meta-Analyses (PRISMA) guidelines for transparent reporting. The primary literature search was performed in the PubMed/Medical Literature Analysis and Retrieval System Online (MEDLINE) database for articles published from January 1, 2000, to March 31, 2025. The search strategy used a combination of Medical Subject Headings (MeSH) terms and free-text keywords related to the hippocampus ("hippocamp*", "hippocampal"), volumetric changes ("atrophy", "volume loss", "volumetric", "morphometry", "volumetry", "gray matter volume"), and radiotherapy ("radiotherapy", "radiation therapy", "irradiation", "whole-brain radiotherapy", "WBRT"). The reference lists of relevant articles and recent reviews were also screened to identify additional eligible studies, including those published online ahead of print.

Inclusion and Exclusion Criteria

Inclusion criteria were: (i) original human studies, (ii) patients receiving therapeutic cranial irradiation for primary or metastatic brain tumors or nasopharyngeal cancer, (iii) longitudinal assessment of hippocampal volume using MRI, and (iv) reported quantitative volumetric data (absolute volume or percentage change). Exclusion criteria were: (i) animal or preclinical studies, (ii) review articles, editorials, case reports, or conference abstracts without original quantitative data, (iii) studies lacking baseline or follow-up volumetric measurements, and (iv) studies focusing solely on postoperative changes without RT.

Study Selection and Data Extraction

Two independent reviewers screened titles and abstracts against the eligibility criteria. The full text of potentially relevant articles was then assessed. Any discrepancies were resolved through discussion and consensus with a third reviewer. From each included study, we extracted the following data using a standardized form: first author, publication year, study design, patient population and sample size, RT details (technique, total dose, fractionation), chemotherapy use, MRI methodology (scanner field strength, sequence, segmentation method), time points of assessment, key volumetric outcomes (mean change, dose-volume correlations), and neurocognitive correlates if reported.

Data Synthesis and Analysis

Given the anticipated and observed heterogeneity across studies in patient populations (e.g., tumor types, ages), radiotherapy parameters (dose, fractionation, technique), MRI methodologies (scanner strength, sequences, segmentation software), and timing of follow-up, a formal quantitative meta-analysis was not feasible. Therefore, a narrative synthesis approach was adopted. The statistical findings (e.g., p-values, correlation coefficients, 95% confidence intervals (CIs), odds ratios) are reported as presented in the original studies to illustrate the strength and significance of the observed effects within each study's context. To facilitate comparison, volumetric outcomes are primarily presented as percent change from baseline, as this metric is less sensitive to absolute segmentation differences than raw volumetric measures. When reported by the original authors, radiation doses are discussed in the context of biological effectiveness (e.g., EQD2 with α/β = 2 Gy for late effects). No formal risk-of-bias assessment tool (e.g., Risk Of Bias In Non-randomized Studies - of Interventions (ROBINS-I)) was applied to individual studies due to the narrative synthesis design; however, major methodological limitations (e.g., small sample size, retrospective design, lack of control group) are highlighted in the text and Table 1 where relevant. The extracted data were tabulated (see Table 1), and studies were grouped thematically for presentation based on the clinical scenario: (i) adult patients with local brain irradiation for CNS tumors, (ii) adult patients with local irradiation for nasopharyngeal cancer, (iii) whole-brain radiotherapy in adults, and (iv) cranial irradiation in children. Within each group, findings are summarized with emphasis on the magnitude and dynamics of volume loss, dose-response relationships, and influencing factors.

**Table 1:** Summary of key studies on hippocampal volume changes after therapeutic cranial irradiation. RT: radiotherapy; WBRT: whole-brain radiotherapy; HA-WBRT: hippocampal-avoidance whole brain radiotherapy; NPC: nasopharyngeal carcinoma; Chemo:  chemotherapy; ChemoRT: chemoradiotherapy; TMZ: temozolomide; IMRT: intensity-modulated radiotherapy; pts: patients; HC: healthy controls; GM: gray matter; VBM: voxel-based morphometry; GCL: granule cell layer; ML: molecular layer; SUB: subiculum; DG: dentate gyrus; CA: cornu ammonis; SR-L-SM: stratum radiatum-lacunosum-moleculare; GC-ML-DG: granule cell and molecular layer of dentate gyrus; Dmax: maximum dose; Dmean: mean dose; D40%: dose to 40% of volume; EQD2: equivalent dose in 2 Gy fractions; CSI: craniospinal irradiation; SCLC: small cell lung cancer; mo: months; yr: year; fx: fractions

Study (Year)	Design	Population (n)	Radiotherapy Details	Hippocampal Dose	MRI & Segmentation Method	Key Volumetric Findings
Local RT for CNS Tumors (Adults)						
Seibert et al. (2017) [19]	Longitudinal	Primary CNS tumors (52 pts, 79 hippocampi)	Local RT (1.8-2 Gy/fx)	Mean dose: >40 Gy vs <10 Gy (stratified)	3T, T1, Automated (NeuroQuant)	Dose-dependent atrophy: significant volume loss (-5.8% or -0.3 ml, p=0.02) at mean dose >40 Gy; insignificant change (-1.2%, p=0.20) at mean dose <10 Gy. Estimated loss: 0.13% per Gy over one year.
Nagtegaal et al. (2021) [20]	Retrospective	Gliomas (31 pts, 42 hippocampi)	Local RT (50.4-60 Gy) ± Chemo	Not explicitly stated per hippocampus	3T, T1, Automated (CAT12)	Dose-dependent volume loss: 0.16% per Gy and 4.9% per 30 Gy over one year. Volume reduction equated to a median "aging" of the hippocampus by 11 years.
Prust et al. (2015) [21]	Prospective longitudinal	Glioblastoma (14 pts)	ChemoRT (60 Gy) + TMZ	Presumably low (contralateral hippocampus assessed).	3T, Multi-modal, Manual (ITK-SNAP)	No significant volume reduction found in the hippocampus contralateral to the tumor over 6 months of follow-up.
Le Fèvre et al. (2021) [22]	Retrospective	Glioblastoma (49 pts)	ChemoRT (60 Gy) + TMZ	D40% (dose to 40% of volume): range from <7.4 Gy to >50 Gy.	3T, Manual contouring	Strongly nonlinear dose effect: volume change per Gray was +2.7% at D40% <7.4 Gy, -0.44% for 7.4-30 Gy, -1.13% for 30-50 Gy, and -5.55% for ≥50 Gy. Significant correlations with Dmax, D98%, D40%.
McDonald et al. (2024) [23]	Retrospective longitudinal	WHO grade 2 glioma survivors (105 pts)	RT (52.9 ±4.3 Gy) vs no RT	Not specified	Automated (SynthSeg)	Radiotherapy exposure independently associated with steeper hippocampal volume decline. At 2 years, patients receiving RT had greater volume loss (-3.1% vs +0.5%, p=0.041). Resection and chemotherapy were also independent factors.
Local RT for NPC (Adults)						
Shi et al. (2018) [24]	Cross-sectional	NPC: Pre-RT (56) vs Post-CRT (40)	IMRT (68.8/70.4 Gy) + Chemo	Dmean: 18.3±9.1 Gy (left head), 21.3±11 Gy (right head).	3T, T1, VBM (SPM8)	Significantly lower gray matter volume in the left hippocampus in the post-RT group compared to pre-RT (voxel-wise analysis, P<0.001, AlphaSim corrected). Hippocampal mean dose was higher than to other structures.
Lv et al. (2019) [25]	Longitudinal	NPC (58) vs HC (20)	IMRT (68-70 Gy) + Chemo	Dmean: 20.4±6.7 Gy (left), 20.6±7.5 Gy (right).	3T, T1, Automated subfields (FreeSurfer)	Time-dependent bilateral volume reduction in total hippocampus, GCL, CA1, ML, and SUB at 3 and 6 months post-RT (p-values from 6.359e-7 to 9.133e-4). Volume loss in these regions correlated with ipsilateral hippocampal dose.
Olsson et al. (2012) [26]	Case-control	NPC survivors (15) vs HC (15)	Curative RT for head and neck cancer	Estimated dose range: 1.5 to 9.3 Gy (low incidental exposure).	1.5T, Manual (custom Matlab)	No significant differences in total, left, or right hippocampal volumes between patients and controls (for total volume: mean difference +37.4 mm³, 95% CI: -357.5 to +432.5, p=0.84).
Li et al. (2025) [27]	Prospective	NPC (193) vs HC (20)	IMRT + Chemo	Dmean: 13.2±5.9 Gy (left), 12.5±6.1 Gy (right).	3T, multi-parametric	Rapid hippocampal atrophy (-5.25%) during the first 3 months (acute phase), followed by relative stabilization up to 12 months. Microstructural changes followed a similar pattern. Dmax identified as primary dosimetric predictor. D50 (50% risk of atrophy): 44.26 Gy (with neoadjuvant chemo) vs 55.06 Gy (without).
WBRT in Adults (Therapeutic & Prophylactic)						
Takeshita et al. (2020) [10]	Longitudinal	Lung CA: WBRT+Chemo (20) vs Chemo only (18)	WBRT (10x3 Gy)	Whole hippocampus received substantial dose (specific metrics not provided).	3T, T1, Automated (FreeSurfer)	Progressive hippocampal volume reduction after WBRT: 1.8% (0-3 mo), 5.8% (4-7 mo), 9.2% (8-11 mo). Mean reduction of 5.7% at ~7.7 months, significantly greater than in cortex or white matter.
Simo et al. (2016) [28]	Longitudinal	SCLC: Prophylactic WBRT+Chemo (22) vs controls (34)	Prophylactic WBRT (25 Gy, 10x2.5 Gy)	Whole brain dose included hippocampi.	3T, VBM (SPM8)	Volume loss in the right hippocampus and parahippocampal gyrus at 3 months post-WBRT (family-wise error corrected p ≤ 0.05 at cluster level).
Popp et al. (2021) [29]	Retrospective longitudinal	Brain mets: HA-WBRT (35) vs WBRT (48)	HA-WBRT (30 Gy/12 fx) vs WBRT (e.g., 35 Gy/14 fx)	Median Dmean (EQD2): ~6.8 Gy (HA-WBRT) vs ~39.4 Gy (WBRT).	Mixed, Automated (CAT)	HA-WBRT significantly reduced hippocampal atrophy: 2-year volume loss was 3.1% vs 8.5% after conventional WBRT. Atrophy progressed over 48 months in both groups but was less severe with HA-WBRT.
de Ruiter et al. (2023) [30]	Randomized (Phase 3)	SCLC: HA-WBRT (57) vs WBRT (46)	HA-WBRT vs WBRT (25 Gy/10 fx)	Mean physical dose in HA-WBRT arm: ≤8.5 Gy.	3T, T1, Automated (FreeSurfer)	HA-WBRT reduced hippocampal atrophy at 4 months (1.8% vs 3.0%) and 12 months (3.0% vs 5.8%) compared to conventional WBRT (significant group-by-time interaction).
Holikova et al. (2024) [31]	Longitudinal	Brain mets: WBRT (10) vs local hypofractionated RT (18)	WBRT (30 Gy/10 fx) vs local RT (e.g., 25 Gy/5 fx)	Whole hippocampus in WBRT field; minimal dose in local RT.	3T, Automated subfields (FreeSurfer)	Significant volume change in the granular cell and molecular layer of the dentate gyrus (GC-ML-DG) subfield of the left hippocampus after WBRT (median change -5 mm³). No significant changes after local hypofractionated RT.
Liu et al. (2025) [32]	Retrospective longitudinal	Brain mets (75)	WBRT (20x2 Gy or 10x3 Gy)	Whole hippocampus in WBRT field.	3T, T1, Manual contouring + Radiomics	Biphasic atrophy: significant volume loss of -1.68% at early follow-up (~26 days) and cumulative loss of -12.51% at long-term follow-up (~393 days). Radiomics changes preceded gross volumetric loss.
Cranial RT (Children)						
Nagel et al. (2004) [33]	Longitudinal	Medulloblastoma (25)	CSI (23.4-39.6 Gy) + boost + Chemo	Whole hippocampus in WBRT field. Posterior regions received higher boost doses.	1.5T, Manual	Hippocampal volumes initially decreased post-treatment, but 2-3 years after diagnosis, growth patterns recovered, though volumes remained smaller. Volume loss was predominant in posterior regions receiving higher boost doses.
Riggs et al. (2014) [34]	Cross-sectional	Pediatric brain tumor survivors (20) vs HC (13)	CSI + Chemo + Surgery	Whole hippocampus in WBRT field. Not specified per hippocampus.	1.5T, T1/DTI, Manual	Smaller right hippocampal volume in survivors compared to controls (p=.03, η²=0.16), independent of total brain tissue volume. In a subset, smaller right hippocampal volume correlated with poorer memory performance.
Decker et al. (2017) [35]	Cross-sectional	Pediatric survivors (29) vs HC (30)	Cranial RT + Chemo	Whole hippocampus in WBRT field. Not specified per hippocampus.	Automated (MAGeT Brain)	Significantly smaller volumes in DG-CA4, CA1, CA2-3, and stratum radiatum-lacunosum-moleculare subfields in survivors compared to healthy controls.

Results

Adult Patients and Local RT of CNS Tumors

Several studies investigated hippocampal volume changes after focal RT for primary CNS tumors, primarily gliomas. 

The study by Seibert et al. included 52 patients who received local RT for primary CNS tumors (mainly gliomas of various WHO grades) [19]. Radiation was delivered in a standard fractionation regimen with a dose per fraction of 1.8-2 Gy. Forty-two patients received 30 fractions. For the remaining 10 patients, doses were converted to the biological equivalent of 30 fractions using an α/β ratio of 2. 3D MRI scans were acquired on a 3 T scanner before or at the start of RT, and 9-15 months after completion. For automatic hippocampal segmentation, T1-weighted non-contrast-enhanced MR images were processed using specialized software (NeuroQuant; CorTechs Labs, Inc., San Diego, California, India) [36]. Hippocampi located within 5 mm of the tumor or postoperative bed were excluded. The final analysis included 79 hippocampi. A significant negative correlation was observed between the mean radiation dose to the hippocampus and the percent volume change at one year (r = -0.24, p = 0.03). Hippocampi receiving a mean dose >40 Gy showed significant atrophy (-5.8% or -0.3 ml, p=0.02), while those receiving <10 Gy did not (-1.2%, p=0.20). A linear mixed-effects model estimated an average volume loss of 0.13% per Gy over one year. In multivariate analysis, both radiation dose and patient age were significant independent predictors of hippocampal atrophy (p<0.01 for both), whereas hemisphere, sex, seizure history, bevacizumab use, and baseline volume were not.

Nagtegaal et al. conducted a retrospective study of 31 patients receiving RT for gliomas (WHO grade II-IV) [20]. Radiation was delivered in conventional fractionation to a total dose of 50.4-60 Gy. Some patients also received chemotherapy (temozolomide - 67.7% or procarbazine, lomustine, and vincristine - 12.9%). MRI, including 3D sequences, was performed on a 3 T scanner as part of standard care. The baseline scan and the scan closest to one year after starting RT were analyzed (median follow-up 319 days, range 270-360). Automatic segmentation of hippocampal and other deep grey matter volumes was performed using the Computational Anatomy Toolbox (CAT12) with the Neuromorphometrics atlas. Hippocampi within the planning target volume were excluded due to potential segmentation artifacts from tumor-related signal changes. The final analysis included 42 hippocampi. A significant dose-dependent volume loss was observed for the hippocampus (p < 0.05, family-wise error rate corrected) with a loss rate of 0.16% per Gy and 4.9% per 30 Gy. Significant volume decreases were also noted in other deep grey matter structures (amygdala, nucleus accumbens, globus pallidus, putamen, thalamus), but not the caudate nucleus. A sensitivity analysis found no clear effect of chemotherapy. In 22 patients aged 52-72 years, comparison with nomograms from the UK Biobank revealed an increased "hippocampal age" after RT, with a median increase of 11 years (range 2-20 years).

Prust and colleagues conducted a prospective longitudinal study to assess volume changes in brain structures, including the hippocampus, in 14 patients with newly diagnosed glioblastoma treated with standard CRT (temozolomide + 60 Gy focal RT) followed by adjuvant temozolomide [21]. Multi-modal MRI (including 3D and DTI) was performed on a 3 T scanner before CRT, weekly during treatment, and monthly during six months of chemotherapy. Manual hippocampal segmentation was performed using ITK-SNAP (https://www.itksnap.org/pmwiki/pmwiki.php), with co-registration of serial scans. The authors assessed the hippocampus contralateral to the tumor (or on the side with lesser involvement). Overall, 14 hippocampi were assessed longitudinally, and no significant volume reduction was found.

Le Fèvre and colleagues retrospectively evaluated hippocampal volumes in patients treated for glioblastoma with surgery, CRT (60 Gy in 30 fractions), and adjuvant temozolomide [22]. They assessed volumes at baseline, at recurrence (median 4.6 months after RT), and at the last available MRI (median 17.6 months after RT). All MRIs were performed on a 3 T scanner. Contouring was performed manually by an experienced radiation oncologist using established atlases [37,38], verified by a second specialist. Hippocampi damaged by tumor or surgery were excluded. Volumes for 98, 96, and 82 hippocampi were assessed at the three time points in 49 patients. The hippocampal volume on the affected side was significantly smaller than on the contralateral side at all time points. The authors suggested this resulted from the tumor/surgery effects or compensatory enlargement of the contralateral hippocampus. The total hippocampal volume at the last MRI was significantly smaller than at baseline. Volumes at recurrence and baseline did not differ significantly. The volume decrease from baseline to the last MRI was 520 mm³ (-17.6%) on the affected side and 190 mm³ (-5.37%) on the contralateral side. The median reduction in hippocampal volume over the entire observation period (median 17.5 months) was 0.33% per month, but it was 5.55% per month for doses above 50 Gy. Significant correlations were found between volume reduction (both sides combined) and Dmax (p=0.001), D98% (p=0.028), and D40% (p=0.0002). For D40%, hippocampi decreased by 4 mm³ per Gy over the entire period. Dose stratification revealed strongly nonlinear dynamics: the hippocampal volume change per Gray was +94.3 mm³ (+2.7 %/Gy) for doses below 7.4 Gy, −8.6 mm³ (-0.44 %/Gy) for doses from 7.4 Gy to less than 30 Gy, −44.5 mm³ (-1.13 %/Gy) for doses from 30 Gy to less than 50 Gy, and −112.2 mm³ (-5.55 %/Gy) for doses of 50 Gy or higher. Notably, an increase in volume was observed at D40% <7.4 Gy.

McDonald and colleagues conducted a large retrospective longitudinal study of 105 survivors of WHO grade 2 glioma, comparing those who received RT (n=61) to those who did not (n=44), with a total of 1974 MRI time points analyzed over a median follow-up of 4.6 years [23]. In the cohort, tumor resection was performed in 79 patients (75.2%), RT (with a mean radiation dose of 52.9 Gy (±4.3 Gy)) was administered to 61 patients (58.1%), and chemotherapy (typically temozolomide) was administered to 66 patients (62.9%). Using automated segmentation (SynthSeg (https://surfer.nmr.mgh.harvard.edu/fswiki/SynthSeg)), they assessed volumes of contralateral cortical gray matter, white matter, and hippocampus. Hierarchical linear modeling confirmed that RT exposure was independently associated with a steeper decline in the volume of all three contralateral structures. For the hippocampus specifically, the slope of volume loss was significantly associated not only with RT (β = −0.0589, p < 0.001) but also with tumor resection (β = −0.069, p < 0.001) and chemotherapy (β = −0.0597, p = 0.003). However, in a subset analysis of patients with available dosimetry data (n=27), the mean radiation dose to the hippocampus was not a statistically significant predictor of the rate of volume loss in the hierarchical linear model (p = 0.106). At the two-year mark, patients who received RT had a significantly greater percent volume loss in the hippocampus (−3.1% vs +0.5%, p = 0.041) compared to those who did not. The study highlights that hippocampal atrophy in glioma survivors is multifactorial, with significant contributions from resection and systemic therapy, alongside the dominant effect of RT.

Adults and Local RT for Nasopharyngeal Cancer

RT for nasopharyngeal carcinoma (NPC) often exposes the medial temporal lobes to significant doses, providing a model to study hippocampal effects of high-dose conformal RT.

Shi et al. compared gray matter volumes (including hippocampus) between 56 patients starting RT for NPC and 40 patients who had received CRT (intensity-modulated radiation therapy, 68.8/70.4 Gy) over a year prior [24]. All patients underwent 3T MRI with T1-weighted 3D sequences. Images were processed using the VBM8 toolbox in SPM8 software (https://github.com/spm/spm8). Voxel-wise analysis (two-sample t-test, P<0.001, AlphaSim corrected) revealed significantly lower gray matter volume in the post-RT group in three regions: the left hippocampus, the right pulvinar, and the right middle temporal gyrus. The mean dose (Dmean) to the hippocampi was significantly higher than to other structures (P<0.001). Dmean values were: 18.3±9.1 Gy for the left hippocampal head, 21.3±11 Gy for the right hippocampal head.

Lv and colleagues followed 58 NPC patients and 20 healthy controls in a longitudinal study [25]. RT was delivered to 68-70 Gy in 30-33 fractions with concurrent chemotherapy (cisplatin/nedaplatin or paclitaxel). Neoadjuvant/adjuvant chemotherapy was also used. Only three patients (5.2%) received RT alone. 3D MRI (3D-BRAVO) was performed on a 3T scanner at baseline, three, and six months for patients, and in parallel for controls. Hippocampal subfield segmentation was performed automatically using FreeSurfer, analyzing seven fields (GCL, CA4, CA2/3, CA1, ML, hippocampal tail, and the SUB). The average dose to the right hippocampus was 20.6±7.5 Gy, and to the left hippocampus was 20.4±6.7 Gy. Significant, time-dependent bilateral volume reduction was observed in the total hippocampus (left: p = 6.359e-7; right: p = 8.335e-9), granule cell layer (GCL) (left: p = 1.550e-4; right: p = 9.133e-4), CA1 (left: p = 7.304e-5; right: p = 2.500e-6), molecular layer (ML) (left: p = 2.060e-6; right: p = 1.491e-8), and subiculum (SUB) (left: p = 4.257e-7; right: p = 6.996e-6) in patients, but not in the control group Thus, different sensitivities of individual parts of the hippocampus were demonstrated. Significant negative correlations were found between the rate of volume loss in the bilateral hippocampus, bilateral GCL, and right ML and the mean dose to the ipsilateral hippocampus (e.g., for right hippocampus: p < 0.001). More pronounced volume loss in these areas, plus the left SUB, correlated with faster cognitive decline (MoCA score). Chemotherapy showed no significant effect.

A retrospective study by Olsson et al. included 15 patients who had received RT for NPC 4-10 years earlier and 15 matched healthy controls [26]. The estimated radiation dose to the hippocampus was low, ranging from 1.5 to 9.3 Gy. MRI was performed on a 1.5 T scanner. Manual segmentation was performed using a custom Matlab routine (Hipposegm) by two blinded raters. The interrater reliability for total hippocampal volume was high (intraclass correlation coefficient, consistency = 0.852). Volumes were normalized to intracranial volume. The mean normalized total hippocampal volume was 4779.3 mm³ in patients and 4741.8 mm³ in controls. No significant differences in hippocampal volumes (total, left, or right) were found between groups (for total volume: mean difference +37.4 mm³, 95% CI: -357.5 to +432.5, p=0.84). The authors concluded that low-dose radiation to the basal brain in adults did not cause a major, lasting volume reduction of the hippocampi.

Li et al. conducted a prospective study with 193 NPC patients (158 discovery, 35 validation cohort) and 20 healthy controls [27]. Participants underwent 3T MRI in 3D and other modes. Beyond volumetry, microstructural changes were assessed using diffusion tensor imaging (DTI), dispersion kurtosis imaging (DKI), and neurite orientation dispersion and density imaging (NODDI). Patients underwent MRI before treatment, in the acute phase (zero to three months post RT, AC), early-delayed (six months post-RT, ED), and late-delayed (12 months post RT, LD) phases. Healthy volunteers underwent parallel scans. Complete four-time-point data were available for 73 discovery and 25 validation cohort patients. Repeated-measures ANCOVA revealed a significant group-by-time interaction for hippocampal volumes (p<0.001), with significant atrophy detected as early as the acute phase compared to baseline, followed by relative stabilization thereafter. A 5.25% hippocampal volume reduction was recorded. There was a fairly rapid atrophy of the hippocampus in the acute period, followed by stabilization during the subsequent phases. Microstructural changes followed a similar pattern. Using a normal tissue complication probability model, the maximum dose to the hippocampus (Dmax) was identified as the primary dosimetric predictor for atrophy at 12 months. The dose associated with a 50% risk of atrophy was 44.26 Gy in patients who received neoadjuvant chemotherapy and 55.06 Gy in those who did not.

WBRT in Adults

WBRT represents a paradigm where both hippocampi receive substantial, albeit often heterogeneous, radiation exposure.

Takeshita et al. compared 20 lung cancer patients receiving WBRT and chemotherapy to 18 receiving chemotherapy alone [10]. WBRT was administered in 10 fractions of 3 Gy. All patients underwent 3D MRI (fast spoiled gradient-echo (FSPGR)) on a 3T scanner at 0-3, 4-7, and 8-11 months after RT. Segmentation was performed in FreeSurfer version 6.0 software (https://surfer.nmr.mgh.harvard.edu/fswiki/rel6downloads) using longitudinal evaluation. The accuracy of segmentation was confirmed by an experienced radiologist. Hippocampal volume reduction was significantly greater in the WBRT group: 1.8%, 5.8%, and 9.2% at 0-3 (n=18), 4-7 (n=11), and 8-11 (n=12) months, respectively, with significant differences between each consecutive time point (0-3 vs. 4-7 months, p=0.02; 4-7 vs. 8-11 months, p=0.01). The mean volume reduction at a follow-up of 6-10 months (mean 7.7 months) was 5.71% (95% CI: 2.81 to 8.61) in the WBRT group, compared to 0.25% (95% CI: -6.05 to 6.55) in the control group (corrected p=0.03). Hippocampal atrophy was significantly more pronounced than that of the cortex (5.7% vs. 1.3%, p=0.01) or white matter (5.7% vs. 1.3%, p=0.02) within the WBRT group. 

Simo et al. studied three groups: 22 small cell lung cancer patients receiving platinum-based chemotherapy and prophylactic WBRT (25 Gy, 10x2.5 Gy), 21 healthy controls, and 13 non-small cell lung cancer patients receiving chemotherapy only [28]. Cognitive tests and 3T MRI (including 3D scans and DTI) were performed at baseline and three months post-treatment. Morphometric analysis was carried out using the longitudinal processing stream in the VBM8 toolbox under the SPM8 software package. Widespread gray matter reductions were observed in the small cell lung cancer patients group after RT, including the right subcortical regions, bilateral insular cortex, and superior temporal gyrus. Critically, volume loss was also significant in the right hippocampus and parahippocampal gyrus (family-wise error corrected p ≤ 0.05 at cluster level). No significant hippocampal volume changes were found in either control group.

Popp et al. conducted a retrospective longitudinal study comparing 35 patients who received HA-WBRT and 48 who received standard WBRT without hippocampal sparing [29]. Standard WBRT was typically 35 Gy in 14 fractions or 30 Gy in 10 fractions (with other fractionations used in a minority of cases), while HA-WBRT was delivered as 30 Gy in 12 fractions according to the HIPPORAD (Hippocampal-avoidance Whole-Brain Radiotherapy with Dose Escalation on metastases) trial protocol, with strict hippocampal dose constraints: D98% ≤ 9 Gy, D2% ≤ 17 Gy, and an aim for Dmean ≤ 10 Gy. The study included 544 MRI scans performed within 24 months before and 48 months after RT. Segmentation used the computational anatomy toolbox (CAT), verified by a neuroradiologist. Dmax and Dmean were converted to EQD2 (α/β=2). For the conventional WBRT group, the median hippocampal Dmean (EQD2) was 39.4 Gy (range 37.5-40.0 Gy). In the HA-WBRT group, the median Dmean was 6.8 Gy for the left hippocampus (range 5.8-8.4 Gy) and 6.7 Gy for the right (range 5.5-9.2 Gy). Hippocampal atrophy was detected after two years in both groups, but was three times less severe after HA-WBRT (3.1% vs. 8.5%). The time course of atrophy was quantified as follows for WBRT vs. HA-WBRT: at six months: -3.0% (95% prediction interval: -7.8 to +1.8%) vs. -0.7% (-4.2 to +2.8%); at 12 months: -5.1% (-10.0 to -0.1%) vs. -1.5% (-5.0 to +2.1%); at 24 months: -8.5% (-13.9 to -3.1%) vs. -3.1% (-6.8 to +0.6%); and at 48 months: -13.8% (-24.7 to -2.9%) vs. -5.2% (-9.7 to -0.7%).

Hong et al., as a part of a larger study, assessed hippocampal volume changes after WBRT for melanoma metastases with (n=4) or without (n=9) hippocampal avoidance, compared to an observation group (n=7) [39]. Data suggested greater volume decrease from baseline to six months in the combined WBRT groups (-5.36%) compared to the observation group (0%). HA-WBRT tended to preserve volume (mean change +0.16%) compared to non-HA-WBRT (-7.81%). No statistical analysis was performed due to the small sample size.

Holikova et al. included 28 patients with brain metastases receiving either WBRT (30 Gy in 10 fractions, n=10) or local hypofractionated RT (most commonly 25 Gy in five fractions or 24 Gy in three fractions, n=18) [31]. A 3T MRI was performed before and four months after RT. Automated hippocampal subfield segmentation was performed using FreeSurfer. Manual segmentation was also done in Eclipse™ software for RT planning (The Eclipse Foundation, Brussels, Belgium). FreeSurfer-derived whole hippocampal volumes were significantly larger than manually contoured ones (p<0.001). No significant volume changes were found in the right hippocampus or in the SRT control group. Significant changes were found in the left hippocampus, specifically in the granular cell and molecular layer of the dentate gyrus (GC-ML-DG) subfield after WBRT (median change -5 mm³). No changes were detected in the local RT group.

De Ruiter and colleagues reported findings from their prospective, multicenter randomized phase 3 trial, directly comparing prophylactic HA-WBRT to conventional prophylactic WBRT in patients with small-cell lung cancer without brain metastases [30]. In the HA-WBRT arm (n=57 at four months, n=28 at 12 months), strict hippocampal constraints were applied: mean physical dose ≤8.5 Gy (biological equivalent ≤6.1 Gy, α/β=2 Gy) and D1% ≤10 Gy. The control arm received standard WBRT with 25 Gy delivered in 10 fractions (n=46 at four months, n=27 at 12 months). Research-quality 3D T1-weighted MRI scans were acquired at baseline, four, and 12 months. Hippocampal volumetry was performed using the longitudinal pipeline of FreeSurfer 6.0. A repeated-measures general linear model revealed a significant group-by-time interaction for hippocampal volume. At four months, the HA-WBRT group showed significantly less volume loss than the conventional WBRT group (-67 mm³, 95% CI -95 to -40 mm³, vs -116 mm³, 95% CI -156 to -77 mm³), corresponding to a 1.8% versus 3.0% reduction (p=0.037 for interaction). This protective effect persisted at 12 months, with volume losses of -115 mm³ (3.0%) and -215 mm³ (5.8%) for HA-WBRT and conventional WBRT, respectively (p=0.002 for interaction). Notably, despite the significant between-group difference, hippocampal atrophy still occurred in the HA-WBRT arm, which received a mean dose of 8.0 Gy. Crucially, no significant associations were found between the magnitude of hippocampal atrophy (or other MRI indices of injury) and decline in memory performance as measured by the Hopkins Verbal Learning Test-Revised.

Shang and colleagues conducted a single-center randomized trial comparing conventional WBRT to hippocampal-avoidance WBRT in 40 patients with brain metastases with a follow-up period of 12 months post-RT [40]. The total prescribed dose was 30 Gy in 10 fractions. In the HА-WBRT arm, planning was performed using hippocampal dose constraints per the RTOG 0933 protocol (D40% < 7.3 Gy, Dmean < 10 Gy, Dmax < 17 Gy. Hippocampal volumes were assessed on T1-weighted images from a 3.0 T MRI scanner, with manual contouring performed by a radiation oncologist following a detailed anatomical atlas. The authors reported that post-treatment hippocampal volume was significantly larger in the HА-WBRT group compared to the conventional WBRT group (p < 0.05). Specific volumetric data were not provided.

A retrospective longitudinal study by Liu et al. quantitatively assessed hippocampal volume changes in 75 patients receiving WBRT (20x2 Gy or 10x3 Gy) for brain metastases using manual contouring on 3.0 T MRI T1-weighted images [32]. The mean hippocampal volume was 3.41 ± 0.49 cm³ before RT. A significant biphasic atrophy was observed: volume decreased to 3.32 ± 0.49 cm³ at the early post-WBRT scan (mean 26 days), representing a 1.68% reduction (p<0.05), and further declined to 2.95 ± 0.45 cm³ at long-term follow-up (mean 393 days), corresponding to a cumulative 12.51% loss compared to baseline (p<0.05). In addition to volumetry, the authors performed a multisequence MRI radiomics analysis. This analysis revealed that changes in numerous quantitative imaging features (radiomics) were more pronounced and occurred earlier than gross volumetric loss. Furthermore, the temporal patterns of these feature alterations differed across MRI sequences, suggesting distinct underlying microstructural injuries evolving over time post-irradiation.

WBRT in Children

The developing brain of children is particularly vulnerable to radiation. Studies in pediatric populations reveal distinct patterns of hippocampal injury and recovery.

Nagel et al. examined 25 children treated with CSI and chemotherapy for medulloblastoma [33]. Mean age at diagnosis was 8.27 years. CSI doses were 36-39.6 Gy (high-risk) or 23.4 Gy (average-risk). Additionally, a boost was performed on the bed and tumor remnants up to a total dose of 55.8 Gy. This was followed by four cycles of cyclophosphamide, cisplatin, and vincristine chemotherapy. Volumetric MRI on a 1.5 T scanner was performed serially for five years post treatment (average 6.36 scans/patient). Hippocampi were manually delineated by a blinded investigator. The authors determined that both hippocampi initially decreased in size after treatment, but two to three years after diagnosis, the structures returned to their normal growth pattern. Notably, the total cranial radiation dose (as defined by risk group) was not a statistically significant predictor of overall hippocampal volume change in the models. Volume loss was predominant in the posterior regions, corresponding to the high-dose boost area. Female sex, low parental education, shunt placement, and seizure history negatively impacted hippocampal volume. The authors presented a significant quadratic function of time since diagnosis that described the dynamics of hippocampal volumes. The predictive models were the following: Left hippocampal volume = 2.6037 - 0.0846t + 0.0197t2. Right hippocampal volume = 2.5643 - 0.0928t + 0.0246 t2, where “t” is time.

Decker et al. assessed hippocampal subfield volumes in 29 pediatric brain tumor survivors treated with cranial radiation and chemotherapy, and 30 healthy controls [35]. The fields were segmented using the multiple automatically generated templates for different brains automated segmentation algorithm. The study showed significantly smaller volumes in DG-CA4, CA1, CA2-3, and stratum radiatum-lacunosum-moleculare in pediatric brain tumor survivors.

Riggs et al. studied 20 pediatric brain tumor survivors (19 medulloblastoma, one astrocytoma) treated with surgery, chemotherapy, and CSI, and 13 healthy volunteers [34]. The mean time from diagnosis to study was approximately five years (range 1.1-11.6 years). MRI was performed on a 1.5 T scanner and included 3D images and diffusion tensor imaging. Manual hippocampal segmentation with multiplanar verification was used, and volumes were corrected for total intracranial volume using a regression-based technique to control for overall brain size. Compared to controls, survivors exhibited significantly reduced total white matter volume (p = .0001; η² = 0.45), lower fractional anisotropy (FA) in the bilateral uncinate fasciculus (p = .03; η² = 0.15), and a smaller right hippocampus (p = .03; η² = 0.16). No significant group differences were found for total gray matter volume (p = .30) or for the left hippocampus (p = .30). Crucially, the reduction in right hippocampal volume remained significant even after controlling for differences in total brain tissue volume (p = .03), indicating a specific vulnerability of the hippocampus beyond global brain atrophy. In a subset of survivors (n=10) who underwent neuropsychological testing, smaller right hippocampal volume and lower fractional anisotropy of the left uncinate fasciculus correlated with poorer performance on a general memory index.

Nieman and colleagues conducted a longitudinal study evaluating brain anatomy in 19 pediatric patients (stratified by age at diagnosis: ≤7 years, n=11; ≥8 years, n=8) treated with cranial radiation therapy for brain tumors, compared to 41 controls [41]. The majority of patients received CSI (23.4 Gy for most) with a boost to the posterior fossa (total dose 54-59.4 Gy). The study assessed total hippocampal volume, among other structures, though the specific segmentation method was not detailed in the context of hippocampal volumetry. Longitudinal analysis using a linear mixed-effects model revealed that, while no significant difference in absolute hippocampal volume was found at the first post-treatment scan, there was a significant negative effect of time since RT. The model estimated a relative decrease in hippocampal volume of 99 mm³/year (approximately 3% per year) in irradiated patients, compared to a growth of 28 mm³/year in controls.

Discussion

This review consolidates substantial data demonstrating volumetric changes in the hippocampus following radiation exposure during RT for CNS tumors (mainly gliomas, medulloblastomas, metastases) and nasopharyngeal cancer. Studies included both pediatric and adult populations, who appear to tolerate exposure differently. Modalities included partial lateralized RT, WBRT, and combinations. Chemotherapy, often administered concurrently, may also influence the process. 

Fractionation Scheme 

This review primarily summarizes data from studies using conventional fractionation (1.8-2.0 Gy per fraction) or moderately hypofractionated regimens (up to 3 Gy per fraction). The impact of stereotactic radiosurgery or hypofractionated RT (>3 Gy per fraction) on hippocampal volume remains largely unexplored, as volumetric studies in such settings are lacking. Theoretically, due to smaller irradiated volumes and steep dose gradients, the effect on global hippocampal volume may be less pronounced [31]. However, emerging cognitive data suggest the hippocampus remains functionally vulnerable. For example, in Gamma Knife radiosurgery for benign sellar lesions, hippocampal doses as low as D40% ≥2.5 Gy were associated with cognitive decline, though volumetric changes were not assessed [42]. This highlights a knowledge gap and the need for future studies combining volumetry and neuropsychological testing after high-dose-per-fraction regimens.

Methodological Considerations in Volumetric Assessment

Most studies used 3T MRI, with fewer using 1.5T, and employed various segmentation methods (manual/automatic). The methods used are generally accurate and reproducible [43-45]. At the same time, segmentation errors cannot be excluded, and very small alterations may fall within the margin of error. In addition, due to its complex anatomy, the hippocampus has areas that are difficult to segment, which may be a source of uncertainty, especially when using 1.5 T MRI [26].

Magnitude of Atrophy

The synthesized data robustly demonstrate a consistent and substantial dose-dependent reduction in hippocampal volume following therapeutic irradiation. The mechanisms likely involve disrupted neurogenesis, apoptosis, inflammation, trophic impairment, and vascular dysfunction, with varying predominance over time [5,6]. For focal radiotherapy of CNS tumors, the estimated volume loss ranges from 0.13% to 0.16% per Gy over one year [19,20], with significantly greater nonlinear atrophy (e.g., 5.55% per month) observed at high doses (>50 Gy) [22]. In the context of whole-brain radiotherapy (WBRT), the effect is more pronounced, with volume loss reaching 5-9% within the first year [10,29]. Previously, atrophy was considered a later biomarker than cognitive changes, requiring higher doses. However, some recent studies have shown early changes even at lower doses or involving specific subregions [27,29,30]. Hippocampal-avoidance techniques (HA-WBRT), which reduce the mean dose to approximately 7 Gy, effectively attenuate but do not completely prevent atrophy, with a two-year volume loss of about 3.1% compared to 8.5% after conventional WBRT [29,30]

The magnitude of this radiation-induced atrophy far exceeds the natural rate of hippocampal volume loss due to healthy aging, which is estimated at 0.38-1.12% per year depending on age [46]. In fact, the radiation-induced volume reduction has been equated to an accelerated "aging" of the hippocampus by a median of 11 years [20]. Moreover, the annualized rates of hippocampal atrophy after WBRT often rival or exceed those observed in neurodegenerative conditions such as Alzheimer's disease (~3.5-4.5% per year) [47,48]. This profound effect underscores the high radiosensitivity of the hippocampus and establishes volumetric loss as a significant biomarker of treatment-related neurotoxicity. 

Importantly, this dose-response relationship is not uniform across all dose levels. Several studies report no significant volume reduction at low hippocampal doses [15,19,21,26]. Furthermore, one study noted a paradoxical increase in volume at very low doses (D40% <7.4 Gy), suggesting either a potential stimulating effect or compensatory hypertrophy in response to contralateral dysfunction [22]. This non-linear response underscores the complexity of hippocampal reactions to radiation.

Temporal Dynamics and Population-Specific Patterns

The temporal progression of hippocampal atrophy is not uniform across studies but reveals several key patterns. In adults, volume loss can follow a biphasic trajectory with early subtle reduction and subsequent substantial decline [32], a pattern of rapid initial decrease followed by stabilization [27], or continuous progressive loss [10,29,30].

This highlights a critical difference between adult and pediatric populations: while adults typically exhibit progressive or stabilized atrophy, children may demonstrate a partial recovery of the hippocampal growth trajectory, albeit from a reduced baseline, indicating a greater inherent plasticity or regenerative capacity in the developing brain [33].

Structural Heterogeneity: Laterality and Subfield Vulnerability

Atrophy does not develop uniformly across the hippocampus. Spatial heterogeneity is driven by non-uniform dose distributions, with regions receiving higher boost doses (e.g., posterior hippocampus) showing greater volume loss [33].Nonuniform irradiation can be expected to result in nonuniform atrophy. Furthermore, studies suggest a potential differential vulnerability between the left and right hippocampi, though findings are not entirely consistent, with some reports highlighting the left [24,31] and others the right hippocampus [34]. It is worth noting that several studies have shown the greater functional significance of the left hippocampus, and it is recommended to prioritize it when limiting doses. Given its more pronounced reduction shown in two out of the three studies, its greater sensitivity can be assumed, although its greater functional significance cannot be ruled out [22,49,50].

Different hippocampal subfields also exhibit varying sensitivity. Studies show specific vulnerability of the DG-CA4, CA1, CA2-3, GC-ML-DG, ML, and SUB regions [25,31,35]. The DG is the part where neurogenesis occurs and is presumably sensitive to radiation exposure because of that fact [51]. The CA1 subregion is highly sensitive to vascular and hypoxic factors, which may also play a role in the effects of radiation exposure.

Modulating Factors Beyond Radiation Dose

While radiation dose is the principal driver, several patient- and treatment-related factors modulate hippocampal atrophy. Advanced patient age is consistently associated with greater volume loss [19]. The impact of systemic therapy is complex and context-dependent. Some studies in nasopharyngeal carcinoma found no clear effect of concurrent chemotherapy [20,25]. Others in glioma survivors have identified chemotherapy (particularly temozolomide) and surgical resection as independent factors associated with hippocampal volume loss, suggesting a multifactorial etiology in multimodal neuro-oncology care [23]. Also, Li et al. found that neoadjuvant chemotherapy increased radiosensitivity [27]. In children, female sex, low parental education, shunt placement, and seizure history were negative factors [33]. This underscores the complexity of disentangling treatment-related effects and highlights the need for comprehensive volumetric assessment in all patients undergoing multimodal therapy.

Clinicoradiological Correlation

The relationship between hippocampal volume loss and cognitive decline is a critical yet complex aspect of radiation-induced neurotoxicity. The evidence presents a nuanced picture, with studies reporting both significant correlations and a lack thereof. For instance, in patients with nasopharyngeal carcinoma, more pronounced volume loss in specific hippocampal subfields correlated with faster decline on the MoCA test [25]. Similarly, in pediatric brain tumor survivors, a smaller right hippocampal volume was associated with poorer performance on a general memory index [34].

However, other well-designed studies, including the randomized trial by de Ruiter et al., found no significant association between the magnitude of hippocampal atrophy and decline in standardized verbal memory tests (HVLT-R) following HA-WBRT [30]. This discrepancy may be attributed to several factors, including differences in the cognitive domains tested, timing of assessments, sensitivity of neuropsychological instruments, patient populations, and the influence of concomitant treatments (e.g., chemotherapy, surgery) that independently affect cognition.

Collectively, these findings challenge a simplistic, direct cause-and-effect model and indicate that isolated hippocampal volumetry may not be a standalone predictor of cognitive outcome in all clinical scenarios. Other radiation-induced pathologies, such as diffuse white matter damage, microstructural injury, and accelerated brain aging, likely contribute substantially to cognitive sequelae, underscoring the necessity of a multimodal biomarker approach for comprehensive risk assessment [30].

Clinical Implications and Future Directions

The consistent finding of dose-dependent hippocampal atrophy underscores the importance of integrating hippocampal protection strategies into modern RT planning. Techniques such as hippocampal-avoidance WBRT have proven effective in significantly reducing volume loss and are now a standard of care in eligible patients. For focal RT, dose constraints to the hippocampus (e.g., D40% < 7-9 Gy) should be considered, especially when treating near the medial temporal lobes. Quantitative hippocampal volumetry can serve as an objective biomarker for treatment-related neurotoxicity in clinical trials and long-term follow-up.

Future research should focus on: establishing definitive dose-volume constraints for the hippocampus across different fractionation schemes, including radiosurgery and hypofractionation; elucidating the differential vulnerability of hippocampal subfields through high-resolution imaging; conducting longitudinal studies that combine advanced MRI (volumetry, diffusion, functional connectivity) with detailed neuropsychological testing to bridge the gap between structural damage and cognitive outcome; and exploring radioprotective or regenerative strategies to mitigate hippocampal injury.

## Conclusions

Quantitative assessment of hippocampal volume change is a reliable and objective biomarker of radiation-induced neurotoxicity. Alongside neuropsychological testing, it helps objectify injury, refine tolerance doses, and elucidate the impact of concomitant factors. Since brain MRI is routine in CNS tumor management, these data can be practically utilized for post-treatment hippocampal assessment. The synthesized evidence confirms that hippocampal volume reduction after therapeutic radiation is a pronounced, dose-dependent process, with rates that can rival neurodegenerative diseases. The vulnerability is not uniform, varying by brain region, subfield, and patient age. Future efforts must focus on leveraging volumetric data to optimize personalized RT plans, prioritizing hippocampal protection, and developing interventions to preserve cognitive function by mitigating structural damage, while accounting for concomitant factors like systemic therapy and individual patient characteristics.
